# Membrane-Bound Class III Peroxidases: Unexpected Enzymes with Exciting Functions

**DOI:** 10.3390/ijms19102876

**Published:** 2018-09-21

**Authors:** Sabine Lüthje, Teresa Martinez-Cortes

**Affiliations:** 1 Oxidative Stress and Plant Proteomics Group, Institute for Plant Science and Microbiology, University of Hamburg, Ohnhorststrasse 18, 22609 Hamburg, Germany; 2 Dpto de Biología Animal, Biología Vegetal y Ecología (Lab. Fisiología Vegetal), Facultad de Ciencias—Universidade da Coruña, A Zapateira s/n, 15071 A Coruña, Spain; teresa.mcortes@udc.es

**Keywords:** *Arabidopsis thaliana*, Class III peroxidase, *Medicago truncatula*, microdomains, phylogenetics, plasma membrane, protein–protein interaction, *Oryza sativa*, tonoplast, *Zea mays*

## Abstract

Class III peroxidases are heme-containing proteins of the secretory pathway with a high redundance and versatile functions. Many soluble peroxidases have been characterized in great detail, whereas only a few studies exist on membrane-bound isoenzymes. Membrane localization of class III peroxidases has been demonstrated for tonoplast, plasma membrane and detergent resistant membrane fractions of different plant species. In silico analysis revealed transmembrane domains for about half of the class III peroxidases that are encoded by the maize (*Zea mays*) genome. Similar results have been found for other species like thale-cress (*Arabidopsis thaliana*), barrel medic (*Medicago truncatula*) and rice (*Oryza sativa*). Besides this, soluble peroxidases interact with tonoplast and plasma membranes by protein–protein interaction. The topology, spatiotemporal organization, molecular and biological functions of membrane-bound class III peroxidases are discussed. Besides a function in membrane protection and/or membrane repair, additional functions have been supported by experimental data and phylogenetics.

## 1. Introduction

Classical secretory plant peroxidases (EC 1.11.1.7; class III peroxidases; donor: H_2_O_2_ oxidoreductases) are heme-containing enzymes that belong to the peroxidase-catalase superfamily [1,2]. According to PeroxiBase, as at August 2018 [3], at least 158 class III peroxidases have been identified in the maize (*Zea mays* L.) genome, 155 isoenzymes in the rice (*Oryza sativa* L.) genome, 103 isoenzymes in the barrel medic (*Medicago truncatula* Gaertn. (Gärtner, Joseph)) genome, and 75 isoenzymes in the thale-cress (*Arabidopsis thaliana* (L.) HEYNH (Heynhold, Gustav)) genome. Differences result partially from some unique peroxidase clusters in monocotyledonous plants that were not found in dicotyledonous plants [4]. Additional isoenzymes can be produced by post-transcriptional and post-translational modifications of peroxidase transcripts [5,6].

As high as the number of peroxidases in plant cells is, so are their implications in different functions various [7,8,9]: peroxidases are involved in cell wall modifications like lignification, suberisation, and cross-linking of hydroxyprolin–rich glycoproteins and polysaccharides. They are involved in phytohormone metabolism, senescence, and in several biosynthetic pathways, and fulfill important functions in stress-related processes [8,10,11,12]. Since peroxidases exhibit an almost 1000-fold higher affinity for hydrogen peroxide as catalases and their activities can be modified in the presence of different stress factors, these enzymes play a key role in the detoxification of reactive oxygen species (ROS) [13].

## 2. Membrane-Bound Class III Peroxidases

Evidence for membrane-bound class III peroxidases has been presented for different plant species and tissues on the protein level [14]. Guaiacol peroxidase activities have been detected at thylakoid and peroxisomal membranes, tonoplast and plasma membranes [15,16,17,18,19,20]. Class III peroxidases from tonoplast and plasma membrane (PM) have been partially purified and characterized in more detail [20,21,22]. Further membrane-bound peroxidases have been identified by genomic and proteomic approaches, but still lack biochemical characterization.

A membrane-bound class III peroxidase has been identified in tonoplast of Madagascar periwinkle (*Catharantus* roseus (L.) d.don) leaves [23]. Green fluorescence protein (GFP)-fusion constructs verified localization of CroPrx01 (CrPrx1) at the inner surface of the tonoplast [21].

At the same time, class III peroxidases have been identified in highly enriched PM preparations of maize roots. At least four PM-bound peroxidases (ZmPrx01, ZmPrx66, ZmPrx70 and pmPOX2a) have been partially purified and characterized [22,24]. Depending on the state of development and oxidative stress, further peroxidases have been identified in PM of maize roots [25].

Proteomic and genomic approaches identified PM-bound class III peroxidases in different plant species and tissues; OsPrx95 has been identified in root PM of a salt sensitive rice cultivar [26]. PsPrx13 has been identified in PM of iron deficient pea (*Pisum sativum* L.) roots [27]. AtPrx64 has been shown to interact with Casparian strip formation at the PM [28,29]. AtPrx64 showed high sequence similarity to AtPrx66 as did AtPrx47 [30]. It was shown that AtPrx66 and AtPrx47 were associated with lignification of vessels, whereas AtPrx64 was associated with lignification of sclerenchyma [30,31]. A recent study demonstrated that aluminum tolerance of tobacco (*Nicotiana tabacum* L.) plants was improved by overexpression of a*tprx64* [32].

A localization of class III peroxidases in detergent resistant membranes (DRM) has been demonstrated for PM of barrel medic, maize and sugar beet (*Beta vulgaris* L.) roots [14,33,34]. MtPrx02 was identified in DRM of barrel medic [33]. A precursor of BvPrx12 has been identified in DRM of sugar beet under iron deficiency [34].

## 3. Structure

**Figure 1:**
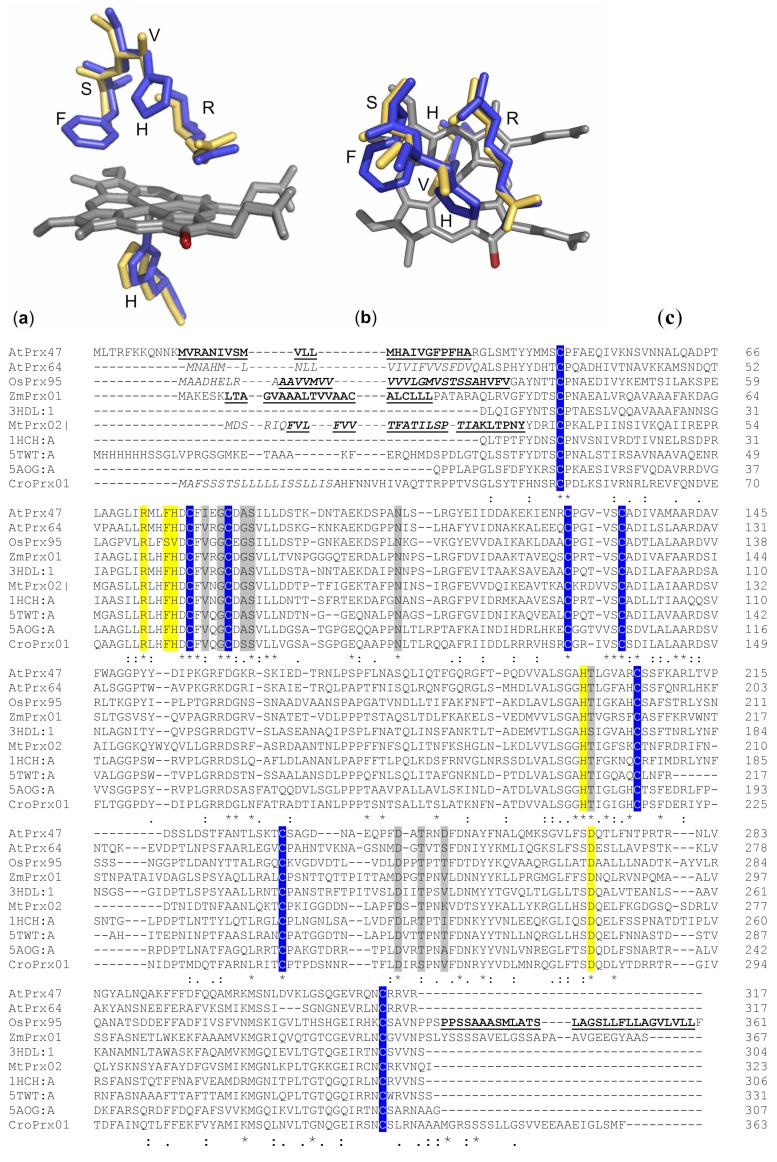
Active sites of horseradish peroxidase (HRP) and OsPrx95 and multiple sequence alignment of class III peroxidases. Superposition of active sites of HRP (blue) and OsPrx95 (yellow) was prepared by Phyton-enhanced molecular graphic tool (PyMOL): (**a**) site view, (**b**) top view; in HRP Arg-38 and His-42 build the distal active-site structure [39]. The heme group (grey), with δ-meso edge (red), is fixed by His-170 in the active site. (**c**) Clustal Omega (https://www.ebi.ac.uk/Tools/msa/clustalo/) was used for multiple sequence alignment of HRP [40], membrane-bound peroxidases and soluble peroxidases that were used as templates for modelling of structures shown in Figure 2 [36,41,42,43]. Active sites (yellow), calcium binding-sites (grey), conserved cysteine residues for formation of disulfide bridges (blue), transmembrane domains (bold and underlined), signal peptides (italic).

The structure of class III peroxidases is well conserved [6]. The proteins contain N-terminal signal peptides, binding-sites for heme and calcium, and four conserved disulfide bridges (Figure 1). Transmembrane domains were predicted for ZmPrx01, OsPrx95, AtPrx47 and MtPrx02, whereas for CroPrx01 and AtPrx64 transmembrane helices (TMH) appear unlikely.

A hypothetical model of CroPrx01 has been published [35]. Besides an N-terminal propeptide (34 amino acids) that directed the GFP-fusion construct of CroPrx01 to the endoplasmic reticulum (ER), a C-terminal extension of 23 to 25 amino acids directed the GFP-fusion constructs of the protein to the vacuole [35]. C-terminal extensions have been found for ZmPrx01 and OsPrx95 (Figure 1). In contrast to CroPrx01, these proteins were identified in highly enriched PM preparations [21,25]. Additionally, prediction of N-glycosylation sites (Table 1) suggested a localization of these enzymes at the apoplastic side.

**Figure 2:**
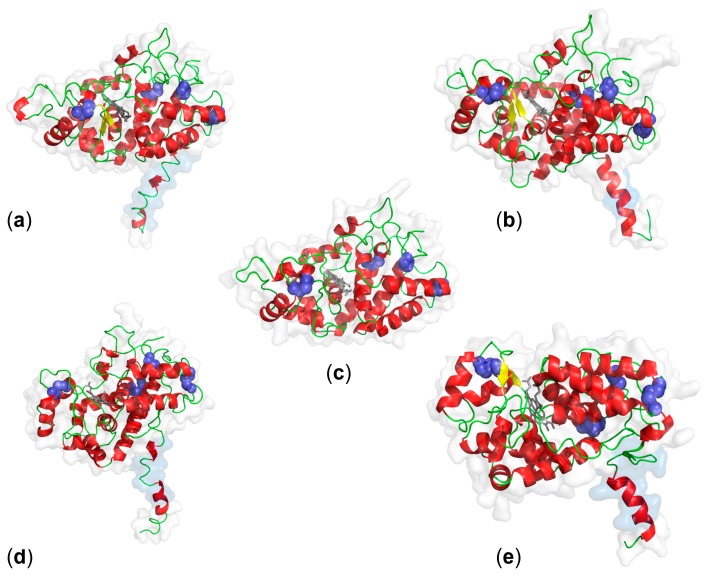
Putative tertiary structures of PM-bound class III peroxidases. Structures were predicted by SWISS-MODEL for (**a**) ZmPrx01 (3hdlA, 1 TMH, root PM), (**b**) OsPrx95 (3hdl1A, 3 TMH, root PM), (**c**) AtPrx64 (3hdl1A, 0 TMH, PM), (**d**) AtPrx47 (5twt, 1 TMH, PM), and (**e**) MtPrx02 (5twt1A, 1 TMH, root tip DRM). N-terminal amino acid sequences containing transmembrane domains (marine) were modeled by ab initio with Phyre2 in intensive mode [49]. Ligand binding-sites were predicted by 3DLigandSite [50]. The hypothetical models were prepared by PyMOL (http://pymol.org/) and present the structural characteristics of class III peroxidases. Colors indicate secondary structures: α-helix (red), β-sheets (yellow), loops (green), disulfides (blue), and haem group (grey).

In accordance with earlier predictions, structures of membrane-bound peroxidases have 13–21 α-helices and between two and 11 β-sheets [14,21]. Exceptions were AtPrx47 and AtPrx64, whose hypothetical structures contained no β-sheets (Figure 2). For AtPrx64, the second calcium binding-site was not conserved (Figure 1). The structures of these peroxidases may need further elucidation.

In comparison to horseradish peroxidase (1HCH1A, HRP) [36], active sites of AtPrx47, AtPrx64, MtPrx02 and ZmPrx01 were well conserved (Figure 1). In contrast, the distal His-42 of HRP was replaced by Val-70 in OsPrx95 (Figure 1). This His facilitates formation of the initial iron-peroxide complex by deprotonating the peroxide and subsequently promote cleavage of the oxygen-oxygen bond by protonating the distal oxygen [37]. In comparison to the wild type HRP, guaiacol peroxidase activity of the H42V mutant was much lower. This observation was in accordance with a ~106-fold lower formation of compound I by the mutant in comparison to the wildtype [38]. As a consequence, reduction of compound II has been more rapid than reduction of compound I. So far, biochemical characterization of OsPrx95 is lacking.

In silico analyzes of putative membrane-bound peroxidases revealed at least one glycosylation-site with the exception of some endoplasmatic reticulum (ER) and vacuolar peroxidases (Table 1). These membrane-bound peroxidases were predicted to be highly phosphorylated. Glycosylation of class III peroxidases seem to be necessary for protein folding, stability and catalytic activity [48]. Glycosylation is necessary for activity of PM-bound peroxidases [22]. Phosphorylation may change properties of the enzymes and be involved in protein-protein interaction. These predictions have to be verified by experimental data.

## 4. Topology and Spatiotemporal Organization

All class III peroxidases appear to have a cleavable signal peptide in common. In silico analyzes of 142 class III peroxidase sequences from maize predicted putative signal peptides, transmembrane domains and the localization of these proteins. This approach revealed a ratio of 53% soluble to 47% membrane-bound isoenzymes [14]. Among the membrane proteins, signals for localization at the endoplasmatic reticulum (ER, 42%), PM (55%), Golgi (2%) and tonoplast (1%) were predicted.

A comparison between rice, barrel medic, arabidopsis and maize showed similar results about the number of isoenzymes with putative transmembrane helices, 51% in rice (*n* = 78), 49% in maize (*n* = 77), 35% in barrel medic (*n* = 36) and 44% in arabidopsis (*n* = 33). The protein subcellular localization prediction tool (PSORT) indicated a PM localization for at least eight class III peroxidases in arabidopsis, 17 in barrel medic, 13 in rice and 27 in maize (Table 1). Additionally, peroxidases were predicted for ER, Golgi, mitochondria outer (MoM) and inner (MiM) membranes. A vacuolar localization was predicted for seven peroxidases in rice and barrel medic, for six in maize, and for one in arabidopsis. A possible localization of these vacuolar peroxidases at the tonoplast will need further proof. For maize roots, peroxidases have been detected at the tonoplast [51].

The properties of PM-bound peroxidases from maize and the identification of class III peroxidases in DRM suggested a tight interaction with the membrane [14,22,24,33,34]. According to membrane-bound ascorbate peroxidases that interact with the membrane via a C-terminal membrane-spanning segment [16,52,53,54,55,56,57,58,59], PM-bound class III peroxidases appeared to be anchored by an uncleavable N-terminal signal peptide with a transmembrane helix. Beyond that, proteomic approaches suggest protein-protein interaction [60]. 

### 4.1. Transmembrane Spanning Domains

In general, N-terminal signal peptides are cleaved off during maturation of soluble secretory peroxidases from eukaryotic organisms. In many cases, the resulting N-terminal positioned glutamine is catalyzed to a cyclic molecule, the so-called pyrrolidone carboxylic acid (PCA) [61]. Prosite predicted such a modification for 32% of the peroxidases shown in Table 1 [62]. Four (OsPrx36, ZmPrx53, ZmPrx78, ZmPrx132) out of 21 vacuolar peroxidases are modified this way potentially. Pyrrolidone carboxylic acid appear absent in CroPrx01 [21] and vacuolar peroxidases from arabidopsis and barrel medic. One third of the putative PM-bound peroxidases showed an N-terminal glutamine that may be modified after cleavage of the signal peptide [21]. 

Three transmembrane helices (TMH) were predicted for OsPrx95 by different prediction tools [14]. PSORT identified a cleavable signal peptide at the N-terminus of OsPrx95 and predicted a type I membrane protein (TMH 345...361, C-terminus) with a localization at the PM (Table 1). This prediction was in accordance with the identification of the protein in the PM [26]. In contrast, the hypothetical model showed a transmembrane domain at the N-terminus (Figure 2). All other peroxidases from Table 1 have one TMH at the N-terminus that is overlapping with the ER signal peptide. Signal peptides of membrane-bound proteins can remain and, due to their hydrophobicity, function as an N-terminal transmembrane domain. Actual search algorithms may be not able to make exact predictions of this possibility yet. So far, crystal structures of membrane-bound peroxidases have not been published.

In accordance with the biochemical classification and with the identification of ZmPrx01, ZmPrx66, and ZmPrx70 at the PM, five of *n* = 5 prediction programs detected N-terminal signal peptides in their amino acid sequences [22]. PSORT even predicted a non-cleavable signal peptide in ZmPrx70 and a localization of the enzyme at the PM or ER membrane (Table 1). The hypothesis of a transmembrane-spanning domain was supported by experimental data: (i) non of the maize peroxidases could be washed off from the PM by high salt concentrations or other treatments [22,24,63]; (ii) peroxidases were found in DRM of washed PM [14]; (iii) recombinant peroxidases were localized in the yeast membrane after heterologous expression of their full length amino acid sequences [64].

### 4.2. Protein–Protein Interaction

Molecular masses of ZmPrx01 (70 kDa) and pmPOX2a (155 kDa) suggested a putative protein–protein interaction [22,24]. Analyzes of the maize PM proteome by high resolution clear native electrophoresis (hrCNE) pointed to the participation of a PM-bound guaiacol peroxidase to a putative high molecular mass protein complex of 1092 kDa [60]. Interaction of AtPrx64 with PM-bound Casparian strip domain proteins (CASPs) was demonstrated [28]. For tonoplast, interaction of CroPrx01 with arabinogalactan proteins (AGPs) was suggested [35].

Protein–protein interaction may be supported by post-translational modifications (Table 1). Phosphorylation may change the properties of peroxidases and facilitate protein–protein interaction, although this hypothesis has to be verified.

### 4.3. Spatiotemporal Organization

**Figure 3:**
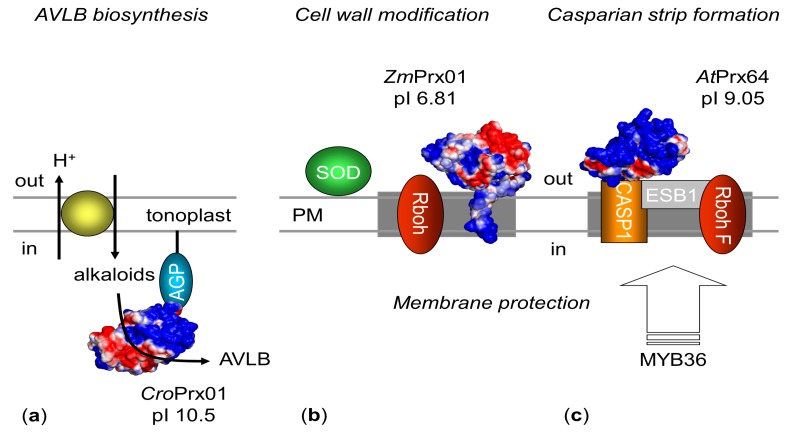
Membrane interaction and spatiotemporal organization of CroPrx01, ZmPrx01 and AtPrx64. (**a**) Soluble CroPrx01 interacts via an arabinogalactan protein (AGP, blue) with the inner surface of the tonoplast [35,67]. For α-3′,4′-anhydrovinblastine (AVLB) synthesis, the complex anchored next to a proton driven alkaloid antiporter (yellow ellipse) [68,69], (**b**) ZmPrx01 is anchored in the PM by a transmembrane-spanning domain with its active site at the out-side [22]. For cell wall modification, the enzyme co-localized with Rboh (ruby colored) in microdomains. Simultaneously, superoxide dismutase (SOD, green) and ZmPrx01 regulate ROS levels at the PM [14,70]. (**c**) Soluble AtPrx64 interact with a transmembrane Casparian strip protein (CASP1, orange) at the PM [28,29,71,72]. A dirigent-like protein (EBS1, grey) facilitates co-localization of the complex with RbohF (ruby colored) in sclerenchyma cells [72]. Casparian strip formation is regulated by MYB36 [29]. For peroxidases solvent accessible surface charges are shown: negative (red); neutral (white), positive (blue).

The proteome of PM microdomains (defined by DRM) is involved in signaling and response to biotic and abiotic stress, cellular trafficking, cell wall synthesis and degradation, and metabolism [65,66]. Evidence for a localization of class III peroxidases in DRM has been presented [14,33,34]. As shown in Figure 3, microcompartimentation allows PM-bound peroxidases to co-localize with ROS producing and detoxifying enzymes in the membrane [25]. Thus PM-bound peroxidases may probably not only detoxify H_2_O_2_ directly at the site of origin to ensure the optimal protection of the membrane, but could also protect specific functional regions of the PM [14,25] and fulfill specific functions in dependence on their substrates.

A co-localization of AtPrx64 with respiratory burst oxidase homolog F (RbohF) has been shown [28]. Besides AtPrx64, RbohF, and a dirigent-like protein (Enhanced Suberin 1, ESB1) were recruited by CASPs to assemble the lignin polymerization machinery [29]. It was shown that this process is regulated by the transcription factor MYB36.

The pattern of CroPrx01:GFP-fusion constructs pointed to localization in mircodomains. A co-localization with AGPs was postulated at the tonoplast [35]. Regulation of CroPrx01 functions were discussed in dependence on the specific localization/orientation.

## 5. Function

Functional analysis and biochemical characterization of membrane-bound class III peroxidases are still fragmentary. It is very difficult to identify the exact function(s) of plant peroxidases because of (i) the huge amount of similar isoenzymes, (ii) the broad substrate specificity, (iii) the high number of possible functions, and (iv) the ability of other isoenzymes to compensate the absence of an enzyme in knock-out experiments [11,73]. Progress has been made by molecular biological approaches in combination with biochemical characterization that resolve some of these problems [74].

### 5.1. Molecular Function

Class III peroxidases are involved in production and scavenging of ROS [7,8,75]. Various substrates are oxidized by the peroxidative cycle that play important functions in polymerization of cell wall components, auxin metabolism, and NAD(P)H oxidation via a non-catalytic reaction. Superoxide anion production via this reaction is immediately converted to hydrogen peroxide and molecular oxygen either spontaneously or through superoxide dismutase. The hydroxylic cycle can produce ROS. Both cycles control hydrogen peroxide levels of cells [25]. A function of hydrogen peroxide in redox regulation and signaling has been demonstrated [76,77].

#### Substrates

The PM is the first target of a stressor. Large amounts of hydrogen peroxide are produced at the PM due to cellular processes, as a response to stress factors, or to external sources in plant–pathogen interactions [78,79,80,81]. This oxidative stress causes lipid peroxidation and changes in membrane permeability [82,83,84]. PM-bound peroxidases were suggested to be substantially involved in the detoxification of the cell and/or repair of the PM after oxidative damage [25]. They probably play an important role in maintaining the cell functions under different stress conditions [22]. 

Class III peroxidases oxidize a wide variety of small phenolic compounds, including monolignols, hydoxycinnamic acids, dimeric alkaloids and others [85,86]. For the docking study shown in Table 2, several of these substrates were tested: (i) monolignols such as coniferyl, sinapyl, and *p*-coumaryl alcohols as precursors of lignin [87,88]; (ii) hydoxycinnamic acids like ferulic acid, caffeic acid and *p*-coumaric acid as precursors of suberin [89]; (iii) L-3,4-dihydroxyphenylalanine (l-DOPA) as precursor of various alkaloids, catecholamines, and melanin [90,91]; and (iv) other substrates like ascorbic acid, indol-3-acetic acid (IAA) and nicotinamide adenine dinucleotides (NAD(P)H). Auxins like IAA have a crucial role in the cell elongation process [92] and can be accumulated in vacuoles [93].

The docking analysis revealed different substrate affinities for the peroxidases in dependence on their localization and biological function. According to their classification, the peroxidases showed low affinity against ascorbic acid (Table 2). 

PM-bound peroxidases of arabidopsis, barrel medic or rice (Figure 1), have not been characterized biochemically yet. For AtPrx64, the substrate affinity decreased in the order NADPH > IAA > NADH > l-DOPA > cinnamyl alcohol > sinapyl alcohol > ferulic acid (Table 2). Gene expression of AtPrx64 indicates that this peroxidase is crucial to lignin deposition in the Casparian strip of endodermis cell walls in roots [28,29]. This observation correlates with affinities of the hypothetical structure of the enzyme against alcohols such as cinnamyl and sinapyl (Table 2). 

The affinity of AtPrx47 decreased from NADPH > IAA > NADH > ferulic acid > cinnamyl alcohol. The docking analyzes revealed that l-DOPA appears not to be a substrate for AtPrx47. These results supported a function of AtPrx47 in the lignification of vessels [30]. 

PM-bound MtPrx02 showed a high affinity for substrates related to cell wall modification, such as IAA, ferulic acid, cinnamyl and sinapyl alcohol (Table 2). This observation confirmed experimental data with this peroxidase [94]. Similar results were found for OsPrx95 that showed high affinity with substrates involved in cell wall modification such as IAA and cinnamyl alcohol (Table 2).

Both, CroPrx01 and AtPrx64, revealed similar affinities with l-DOPA, whereas the other peroxidases showed no reaction with this substrate. This observation excluded a localization of AtPrx47, MtPrx02, OsPrx95 or ZmPrx01 at the tonoplast or in the vacuole. As mentioned above, the structure of AtPrx64 may need further elucidation and results from the docking study have to be verified by biochemical characterization of the native enzyme and/or recombinant polypeptide. Biochemical differences between native proteins and recombinant polypeptides may be possible due to a distinct degree of glycosylation.

Cinnamyl alcohol is easily oxidized by most of the peroxidases, but coniferyl and sinapyl alcohol seem to be poor substrates for many of these enzymes. Thus even during lignin synthesis, sinapyl alcohol dehydrogenation may be mediated by other phenolic radicals [95,96].

### 5.2. Biological Functions

The expression of peroxidase enzymes is regulated and induced by several stressors, such as pathogens, temperature, nutrient starvation or environmental changes [74]. Regulation of peroxidases is strongly effected by ROS levels in the plants. These ROS levels are linkups to the stressors, related besides the self-regulation of the plant [97].

Although class III peroxidases are highly conserved, the high similarity in protein structure has no simple correlation with the functions of these enzymes. Nevertheless, the structural features of class III peroxidases, like distribution of surface charges, glycosylation patterns or even the regulation of the enzymes, seem to be important in the classification by functions of the enzymes [74]. Another feature that was used to characterize peroxidases is the isoelectric point (pI) [6]. So far, little or no information can be found on the correlation between pI and biological functions. 

Both, alkaline and acidic stress, generate an increase of ROS in plant tissues [98,99,100]. Those changes in soil pH and apoplast have a significant effect on the regulation of peroxidases that are linked to ROS generation and scavenging. On the one hand, several studies showed optima in the range between pH 4.5 to 6.5 for peroxidase activity [101,102]. On the other hand, pH is crucial for the stability of peroxidases [103].

Evaluation of Table 1 revealed several membrane peroxidases that respond to pH in rice (*n* = 2) and maize (*n* = 11), but none in arabidopsis or barrel medic. In maize, a localization of these isoenzymes was predicted for PM (*n* = 7), vacuole (*n* = 2) and mitochondria inner membrane (*n* = 2). In rice, two isoenzymes were predicted for PM. All these peroxidases were anionic with the exception of ZmPrx140 (Table 1).

A proteomic approach suggested specific functions of ZmPrx01, ZmPrx66, ZmPrx70 and pmPOX2a in response to oxidative stress [63]. Both, substrate affinity and specific activities make a function of ZmPrx01 in lignification most likely. Several putative cis-regulatory elements were identified by partial gene analysis of ZmPrx70 that suggested a regulation of its gene by wounding, methyl jasmonate, salicylic acid, and elicitors [22]. In addition, a regulation of the gene by oxidative stress was suggested by seven successive elements with sequence similarity to the consensus sequence of antioxidant-responsive elements (ARE) at the 5’-untranslated region of ZmPrx70 [63,104]. Recent studies showed that high-light conditions can increase phenol and peroxidase levels and by that the hydrogen peroxide scavenging capacity of the vacuole. In fact it was proposed that the vacuolar couple CroPrx01/secondary metabolites represent an important sink/buffer of hydrogen peroxide in green plant cells [105].

#### 5.2.1. Orthologous

According to Welinder et al. [61], truly orthologous of peroxidases should have sequence identities greater than 90%. For the peroxidases discussed above, orthologous were not found for the species investigated. Sequence similarities above 70% might indicate related physicochemical properties and physiological functions.

Homologous of AtPrx47 appear not to exist in arabidopsis, highest sequence similarity was 47% (Table 3). For AtPrx47, a function in lignification of vessels has been shown during plant development [29,30]. This observation was supported by the docking analyzes (Table 2). Several proteins with sequence similarities above 80% to AtPrx47 were found in the Brassicaceae family, e.g., AruPrx47 (94.9%) from horseradish (*Armoracia rusticana* G.Gaertn., B.Mey (Bernhard. Meyer). and Scherb. (Scherbius, Johannes)). Sequence similarities above 70% were indicated for MtPrx19 (G7IJT4) and CroPrx09. However, MtPrx19 was suggested to have a function in response to pathogens, whereas the function of CroPrx09 is unkown. For maize and rice, sequence similarities were below 60%. In contrast to AtPrx47, none of the putative orthologous appear to have a transmembrane-spanning domain or a localization at the PM.

Similar results have been found for the tonoplast-bound CroPrx01. Sequence similarity of 84% was found between CroPrx01 and CroPrx03 (B2G335), but in maize, rice, barrel-medic or arabidopsis, sequence similarities were below 60%. CroPrx03 is a soluble apoplastic peroxidase that is expressed in stem and flower tissues [106]. The transcript was down-regulated under salt and dehydration stress.

For the barrel medic MtPrx02, MtPrx70 (G7KFK8) was found as homologous by sequence similarity (Table 3). Indeed this peroxidase responded to nitrogen starvation and pathogen infection. In contrast to MtPrx02, MtPrx70 respond to mycorrhiza and appears to be a soluble peroxidase. PSORT predicted localization outside the cell and the enzyme does not contain a transmembrane helix. Arabidopsis, rice and maize showed similarities below 60% (Table 3).

True orthologous of OsPrx95 were not indicated in maize, barrel medic or arabidopsis. Highest sequence similarity to OsPrx95 was found for ZmPrx94 (A0A1D6HQQ8). Both proteins were predicted at the PM (Table 1). Salt stress caused a decrease in OsPrx95 abundance [27]. In contrast, ZmPrx94 was induced by salinity. The lignin biosynthesis pathway altered significantly under salt stress. For example, cell wall thickness of vessels in sorghum [107] and thickness of Casparian strip in maize roots [108] increased during acclimation to salinity.

Orthologous of ZmPrx01 were not found in rice, Madagascar periwinkle, barrel medic or arabidopsis. In maize, ZmPx101 (B4FU88) and ZmPrx49 (B4FY83) showed highest sequence similarity to ZmPrx01. Both proteins appear to be soluble peroxidases. ZmPrx101 is induced by pathogens, whereas the function of ZmPrx49 is unknown.

Similar results were found by comparison of all ER, PM and vacuolar peroxidases from arabidopsis, barrel medic, rice and maize. Among ER-bound peroxidases, the highest sequence identity was between ZmPrx135 and MtPrx58 (47.33%), the lowest between ZmPrx46 and AtPrx69 (37.15%). In total, 45 putative PM-bound peroxidases were found. The highest sequence identities were found for ZmPrx41 and ZmPrx55 (97.95%) > ZmPrx66 and ZmPrx114 (80.89%) > OsPrx135 and ZmPrx115 (80.43%) > OsPrx30 and ZmPrx123 (79.88%), but for most of them sequence identities were below 60%.

ZmPrx41 responded to pH, drought and etiolation, whereas ZmPrx55 appears to have a function in cold stress. ZmPrx66 showed response to drought, ZmPrx114 appears to have a function in drought stress, but responded also to pathogens and pH (Table 1). ZmPrx115 was affected by etiolation, and is involved in cell wall modification whereas the function of OsPrx135 is unkown. 

For vacuolar peroxidases sequence identities were below 60% with the exception of ZmPrx53 and ZmPrx132 (86.28%). Functions of these peroxidases are unknown.

#### 5.2.2. Phylogeny 

**Figure 4:**
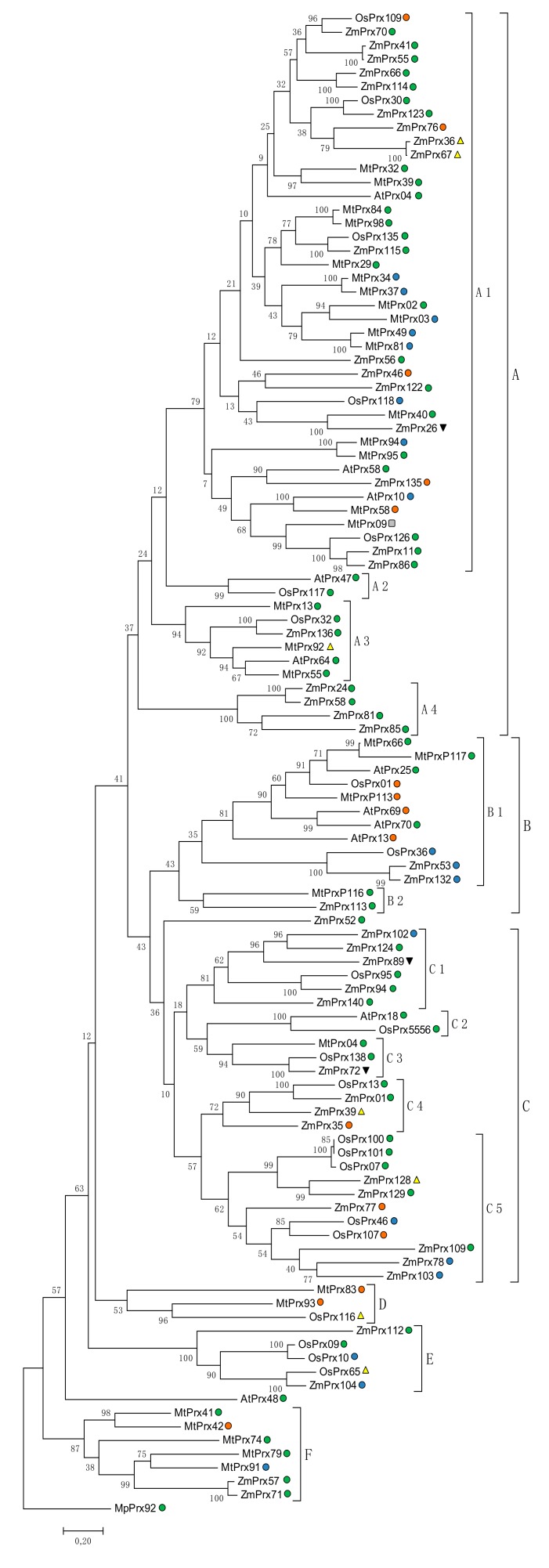
Phylogenetic analysis of peroxidases from Table 1 and MpPrx92 by the maximum likelihood method. The evolutionary history was inferred by using the maximum likelihood method based on the Jones, Taylor, Thornton (JTT) matrix-based model [110]. The tree with the highest log likelihood (-58984.24) is shown. The percentage of trees in which the associated taxa clustered together is shown next to the branches. Initial tree(s) for the heuristic search were obtained automatically by applying Neighbor-Join and BioNJ algorithms to a matrix of pairwise distances estimated using a JTT model, and then selecting the topology with superior log likelihood value. The tree is drawn to scale, with branch lengths measured in the number of substitutions per site. The analysis involved 110 amino acid sequences. There were a total of 841 positions in the final dataset. Evolutionary analyses were conducted by Molecular Evolutionary Genetics Analysis (MEGA7) [111]. ●, ER; ●, PM; ●, Vac; ■ Golgi; ▲, MoM; ▼, MiM.

A phylogenetic tree was performed to obtain further clues to the biological functions of membrane-bound peroxidases in maize, rice, barrel medic and arabidopsis (Figure 4). The phylogenetic tree was constructed using the maximum likelihood method based on the Jones, Taylor, Thornton (JTT) matrix-based model [99]. The analysis was done with amino acid sequences of peroxidases from Table 1. In total 110 sequences were analyzed, Eleven from arabidopsis, 31 from barrel medic, 22 from rice, 45 from maize and one from the liverwort *Marchantia polymorpha* (MpPrx92). The tree was rooted to MpPrx92. The amino acid sequence reveals no signal peptide, it has a prediction for 1 TMH and for a localization of the protein at the PM. Phylogenetic analysis allows identifying the evolutionary conservation and divergence of these enzymes from the common ancestor MpPrx92. 

The tree was divided into six major groups (A to F). The first group, which clusters peroxidases that respond to various stresses, is subdivided in four clusters (A1 to A4). Cluster A1 summarized peroxidases that were characterized to work against several stresses, like drought (e.g., MtPrx29, ZmPrx66 and ZmPrx70), cold (ZmPrx55), pathogens (e.g., MtPrx02, MtPrx32 and ZmPrx86) and even hormones (e.g., AtPrx10 and MtPrx94). Cluster A2 contains only two PM peroxidases that participate in oxidative stress (OsPrx117) and lignification of vessels (AtPrx47). In fact the similarity between the two peroxidases is remarkable (Table 3), but the similarities of biological functions need further proofs. In group A3, peroxidases clustered together that are involved in reactions against pathogens (e.g., AtPrx64, MtPrx55 and MtPrx92) and nodulation (e.g., MtPrx13, MtPrx55 and MtPrx92). Nodulation is characteristic for legumes like barrel medic [109]. Peroxidases from other species in this cluster work most probably in defense against pathogens. Finally, the cluster A4 is formed exclusively by PM peroxidases of maize that are involved against stresses like drought (ZmPrx81) and defense against pathogens (ZmPrx85). 

Group B is subdivided into two clusters but there is not so much known about this group.

Group C is subdivided in five clusters with different responses to abiotic stress. Group C1 contains peroxidases that were not characterized experimentally. A BLAST search suggests a function in response to pH changes, such as soil acidification. This group is formed only by maize peroxidases, with the exception of OsPrx95 from rice. Maize and rice are two of the bigger crops in the world, and frequently the growing of these plants is related to soil conditions. Low pH causes less growing of the plant and even death. So it is very interesting to find peroxidases in these two species that might be involved in this kind of stress. Additionally, this stress can be related to other stresses, like nutrient starvation or drought [112]. The C2 cluster is build by two PM peroxidases that are involved in plant development. AtPrx18 is related to the development of the floral organ and OsPrx5556 could be involved in the response to hormones and heat.

Peroxidases from group C3 seem to be involved in nitrogen starvation response. Three peroxidases of three different species, MtPrx04, OsPrx138 and ZmPrx72, build this cluster and, despite the fact that only one of these peroxidases was studied (MtPrx04), the similarity between them is so high that they may have the same function. This could mean that these peroxidases have a common origin to protect the plant against nutrient deficiency. Cluster C4 is build by peroxidases that are involved in processes like cell wall modification (e.g., ZmPrx01). Finally, group C5 contains peroxidases that are not characterized. A BLAST search suggested a function in response to different abiotic stresses like pH and drought (ZmPrx78) or nitrogen and phosphate starvation (OsPrx46 and OsPrx107).

Group D is formed by three peroxidases from barrel medic and rice that are not characterized.

Group E include mainly peroxidases that are involved in etiolation and are regulated by light and dark (e.g., ZmPrx104 and ZmPrx112). However, these functions are only theoretical because none of these peroxidases were characterized experimentally.

Finally, group F is involved mainly in nodulation (e.g., MtPrx41, MtPrx42 and MtPrx74). In accordance with this specialization of legumes, the majority of peroxidases were from barrel medic. Two peroxidases were from maize (ZmPrx57 and ZmPrx71), and these were separated from the others. The genes showed distinct expression patterns: z*mprx57* has been found in immature leaf, whereas z*mprx71* has been expressed in primary root [113]. Both proteins have a prediction for PM localization. At least one of them may respond in defense against pathogens. This function can be easily related to the nodulation in barrel medic plants. However, biochemical characterization of the maize peroxidases is still missing.

## 6. Conclusions

Molecular biodiversity of membrane-bound class III peroxidases appears to be higher then expected. Although plants evolved the same molecular mechanisms, true orthologues of membrane-bound peroxidases were not identified in the four species investigated. Besides soluble class III peroxidases that interact with the membrane by protein–protein interaction, type I membrane proteins may exist. Membrane-bound peroxidases are multifunctional enzymes that fulfill essential functions in plant development and stress response. Microcompartmentation and co-localization of PM-bound peroxidases with ROS-producing and detoxifying enzymes may probably not only detoxify hydrogen peroxide directly at the site of origin, but could also protect specific functional regions of the PM and fulfill specific functions. Biochemical characterization of membrane-bound peroxidases is still fragmentary and will need further elucidation. Some of these peroxidases may have the potential for marker-assisted breeding.

## Figures and Tables

**Table 1:** Properties and physiological functions of membrane-bound class III peroxidases of arabidopsis, barrel medic, rice and maize. After identification of peroxidase sequences with trans-membrane domains [44], signal peptides [45], post translational modifications [46] and localisation [47] of proteins have been further analysed by bioinformatic tools. In case of missing experimental data on biological functions these have been predicted by a Basic Local Alignment Search Tool (BLAST) (*) search against all peroxidases (inclusive soluble isoenzymes) with highest sequence similarity and known function. ER, endoplasmatic reticulum membrane; Golgi, golgi apparatus; MoM, mitochondrial outer membrane; MS, identified by mass spectrometry [14]; MW, molecular weight; pI, point isoelectric; PM, plasma membrane; PSORT, protein subcellular localization prediction tool; Vac., vacuole. Predicted pyrrolidone carboxylic acid (PCA) modification (+).

Species	Acc. No. ^1^	Protein ^2^	MW ^3^	pI ^3^	SignalP ^4^	N-Glyco ^5^	NetPhos^6^	PCA ^7^	PSORT ^8^	Response to
*A. thaliana*	Q9LE15	AtPrx04	34.28	7.87	1-19	1	39	+	PM	senescence
	Q9FX85	AtPrx10	38.03	6.17	---	2	29	---	Vac	hormones and stress
	O49293	AtPrx13	36.88	5.15	---	1	23	+	ER	---
	Q9SK52	AtPrx18	35.50	5.03	1-29	2	41	---	PM	floral organ development
	O80822	AtPrx25	37.45	8.48	---	2	38	---	PM	etiolation and drought
	Q9SZB9	AtPrx47 ^MS^	35.97	8.57	---	1	30	---	PM	lignification of vessels
	O81755	AtPrx48	44.79	4.99	---	2	42	---	PM	---
	P59120	AtPrx58	35.29	4.92	1-23	2	30	+	PM	---
	Q43872	AtPrx64 ^MS^	34.71	9.05	1-22	3	30	---	PM	etiolation and drought
	Q96511	AtPrx69	35.68	9.47	1-23	3	29	---	ER	etiolation and cold stress
	Q9FMI7	AtPrx70	36.00	6.97	1-23	2	25	---	PM	---
*M. truncatula*	G7KFK6	MtPrx02 ^MS^	36.02	9.20	1-23	1	27	---	PM	nitrogen starvation, wounding and pathogen
	A0A072UYJ4	MtPrx03	35.99	5.35	1-25	3	28	---	Vac	rhizobium inoculation
	G8A179	MtPrx04	35.48	8.32	1-22	5	34	---	PM	nitrogen starvation and pathogen
	A0A072URQ9	MtPrx09	35.92	6.51	1-25	6	41	+	Golgi	phosphate starvation and pathogen
	G7IC23	MtPrx13	35.26	8.01	1-25	2	22	---	PM	nodulation and pathogen
	G7KFM2	MtPrx29	35.79	9.41	1-28	1	27	+	PM	drought, wounding, nitrogen starvation, phosphate starvation, pathogen, methyl jasmonate and ultraviolet (UV) irradiation
	A0A072UJD7	MtPrx32	36.88	8.62	1-23	1	35	---	PM	nitrogen starvation and pathogen
	Q1RXM7	MtPrx34	34.38	9.11	1-22	3	32	---	Vac	drought, insect damage and pathogen
	I3S041	MtPrx37	34.90	7.15	1-22	1	30	---	Vac	drought, insect damage, phosphate starvation and pathogen
	G7IF04	MtPrx39	36.19	6.5	1-25	5	31	---	PM	pathogen
	A0A072UQ08	MtPrx40	37.48	4.73	1-28	2	33	+	PM	pathogen
	A0A072TWY1	MtPrx41	34.60	5.03	1-23	1	30	+	PM	methyl jasmonate, nodulation and pathogen
	Q1SC11	MtPrx42	36.08	9.09	1-25	1	27	+	ER	phosphate starvation and pathogen
	A0A072UHR9	MtPrx49	35.30	9.72	1-25	---	31	---	Vac	nodulation and pathogen
*M. truncatula*	G7I8C1	MtPrx55	34.47	9.45	1-23	2	33	---	PM	nodulation and pathogen
	G7K822	MtPrx58	36.06	6.87	1-25	2	31	+	ER	nodulation and pathogen
	G7JCW8	MtPrx66	34.64	8.21	1-19	1	27	+	PM	nitrogen starvation and pathogen
	A0A072UYL4	MtPrx74	38.49	9.28	1-29	2	28	+	PM	nodulation and pathogen
	I3T3G6	MtPrx79	36.97	8.82	---	2	48	---	PM	nodulation *
	A0A072UIE4	MtPrx81	35.06	8.14	1-25	---	30	---	Vac	pathogen
	G7JZ11	MtPrx83	41.78	5.78	1-23	---	29	---	ER	---
	G7J9S0	MtPrx84	33.82	9.17	1-26	2	44	+	PM	---
	Q1SAT8	MtPrx91	36.52	7.22	1-21	3	35	---	Vac	---
	G7LB60	MtPrx92	34.76	8.81	1-25	1	40	---	MoM	nodulation and pathogen
	G7JJA7	MtPrx93	35.84	5.79	1-23	2	36	+	ER	---
	G7JKU3	MtPrx94	46.33	9.21	---	3	49	---	Vac	methyl jasmonate
	G7JKV1	MtPrx95	38.06	6.14	---	5	36	---	PM	methyl jasmonate *
	G7J9S2	MtPrx98	33.99	8.06	1-26	3	48	+	PM	---
	G7INU9	MtPrx[P]113	12.74	5.56	1-24	---	10	---	ER	cadmium treatment, nodulation, nematode
	A2Q4B7	MtPrx[P]116	33.77	7.98	1-14	1	23	---	PM	infection and pathogen *
	G7JCW9	MtPrx[P]117	17.60	6.23	1-19	---	13	---	PM	nitrogen and phosphate starvation and pathogen *
*O. sativa*	Q5VR15	OsPrx01	34.78	6.88	1-24	1	30	---	ER	---
	Q5U1T6	OsPrx07	31.93	5.32	1-20	1	27	---	PM	---
	Q5U1T4	OsPrx09	35.50	5.67	1-11	5	31	---	PM	etiolation and drought *
	Q5U1T3	OsPrx10	36.20	5.38	---	5	34	---	Vac	---
	Q5U1T0	OsPrx13	36.18	5.16	1-21	8	18	---	PM	---
	Q6ER51	OsPrx30	34.43	7.52	1-26	1	33	+	PM	---
	Q5U1R1	OsPrx32	34.06	8.42	1-30	2	30	---	PM	pathogen *
	Q5U1Q7	OsPrx36	50.76	4.85	1-17	2	45	+	Vac	---
	Q5U1P7	OsPrx46	35.95	7.02	1-29	1	20	---	Vac	nitrogen and phosphate starvation *
	Q7XUL1	OsPrx5556	51.48	9.15	1-26	5	86	+	PM	gibberellic acid and heat *
	Q6AVZ8	OsPrx65	37.61	7.20	1-24	2	21	---	MoM	drought *
	Q8L3W2	OsPrx95 ^MS^	37.58	5.63	1-28	6	38	---	PM	---
	Q6Z121	OsPrx100	32.78	5.68	1-20	2	27	---	PM	---
	Q6Z121	OsPrx101	32.78	5.68	1-20	2	27	---	PM	---
	Q8GVG6	OsPrx106-1	40.15	7.22	1-19	2	47	---	Vac	---
	A2YP47	OsPrx106-2	40.15	7.22	1-19	2	45	---	Vac	---
	Q8GVG0	OsPrx107	34.23	9.19	1-22	3	15	---	ER	nitrogen and phosphate starvation *
	Q5U1I4	OsPrx109	33.02	8.26	1-21	4	32	+	ER	anaerobic stress, brassinolide and giberellic acid treatments and nematode infection *
*O. sativa*	Q6Z3Y8	OsPrx116	36.05	7.69	1-19	1	29	+	MoM	pH and oxidative stress *
	Q5U1H6	OsPrx117	33.51	6.65	1-25	1	30	---	PM	pH, oxidative stress, nitrogen starvation and pathogen *
	Q6UU25	OsPrx118	36.93	4.96	1-25	2	23	---	Vac	---
	Q7XHB1	OsPrx126	35.48	4.45	1-27	1	33	+	PM	pH, oxidative stress, nematode infection and pathogen *
	Q5U1F8	OsPrx135	34.83	8.79	1-31	3	38	+	PM	---
	Q5U1F5	OsPrx138	35.66	8.40	---	2	17	---	PM	nitrogen starvation *
*Z. mays*	A5H8G4	ZmPrx01 ^MS^	38.36	6.81	---	5	35	---	PM	cell wall modification, wounding and pathogen
	A0A1D6KUF1	ZmPrx11	35.44	5.14	1-28	3	28	+	PM	pH, oxidative stress and pathogen *
	B4FHG3	ZmPrx24 ^MS^	37.82	5.9	1-25	2	36	---	PM	pH, oxidative stress, abscissic and ethylene stress, etiolation and heat shock *
	B4FD28	ZmPrx26	37.50	5.01	1-38	3	34	---	MiM	pH, drought and pathogen *
	B6T173	ZmPrx35	36.80	6.05	1-22	2	24	---	ER	---
	A0A1D6H655	ZmPrx36	33.09	4.90	1-24	2	29	+	MoM	heat and oxidative stress *
	B4FVT1	ZmPrx39	37.91	8.30	1-21	8	34	---	MoM	pathogen
	B4FJX1	ZmPrx41	35.75	4.88	---	1	30	---	PM	pH, drought and etiolation *
	A0A1D6FBJ6	ZmPrx46	38.65	6.19	1-23	1	33	---	ER	---
	Q9ZTS6	ZmPrx52	34.59	8.96	1-27	4	29	---	PM	pathogen *
	K7VQB0	ZmPrx53	46.55	4.98	1-24	1	42	+	Vac	---
	B6TU39	ZmPrx55	34.96	4.85	1-42	1	33	+	PM	cold stress
	A0A1D6IMZ0	ZmPrx56	34.57	4.35	1-25	3	36	---	PM	pathogen *
	B6T5R9	ZmPrx57	37.58	5.73	1-19	3	26	---	PM	pathogen *
	B4FH68	ZmPrx58 ^MS^	36.91	6.35	1-25	1	30	---	PM	pH, oxidative stress, abscissic and ethylene stress, etiolation and heat shock *
	A5H454-1	ZmPrx66 ^MS^	33.42	8.39	1-29	4	70	+	PM	drought
	A0A1D6H655	ZmPrx67	32.77	4.89	---	4	26	---	MoM	heat, drought, pathogen and oxidative stress *
	A5H452	ZmPrx70 ^MS^	33.41	9.08	1-25	4	34	+	ER/PM	drought
	B4FMF8	ZmPrx71	35.71	9.64	1-28	3	30	---	PM	---
	B4F7T9	ZmPrx72	36.67	9.31	---	2	22	---	MiM	nitrogen starvation *
	B6SNF9	ZmPrx76	33.30	8.64	1-24	---	33	---	ER	drought
	B4FH35	ZmPrx77	35.71	9.64	1-32	6	20	+	ER	---
	A0A1D6IKW2	ZmPrx78	41.42	5.86	1-21	3	40	+	Vac	pH and drought *
	B4FG39	ZmPrx81	36.42	7.66	1-29	3	35	---	PM	drought
	A0A1D6E530	ZmPrx85	35.48	5.35	1-23	1	32	---	PM	pathogen
*Z. mays*	Q9ZTS8	ZmPrx86	35.51	4.37	1-21	3	34	+	PM	pathogen
	A0A1D6HQQ9	ZmPrx89	36.08	4.87	1-32	10	23	+	MiM	pH, anoxia, ethylene and gibberellic acid *
	A0A1D6HQQ8	ZmPrx94	37.24	5.82	1-29	6	30	---	PM	drought
	B6TWB1	ZmPrx102	37.11	6.00	1-26	4	24	---	Vac	pH and pathogen *
	A0A1D6IKX3	ZmPrx103	57.26	5.06	1-27	4	106	---	Vac	---
	B4FBH0	ZmPrx104	38.04	8.09	1-27	2	23	---	Vac	etiolation *
	B4FYH1	ZmPrx109	37.94	6.00	1-22	3	40	---	PM	pathogen *
	A0A1D6J1L2	ZmPrx112	41.37	7.03	1-27	6	64	---	PM	etiolation, drought and pathogen *
	A0A1D6KQI0	ZmPrx113	38.28	10.26	---	3	27	---	PM	---
	C0PPB6	ZmPrx114	32.71	5.20	1-23	5	38	+	PM	pH, drought and pathogen *
	B6U6W0	ZmPrx115	37.59	8.31	---	3	40	+	PM	etiolation, cell wall modification, wounding and pathogen *
	A0A1D6H652	ZmPrx122	34.37	6.88	1-28	2	29	+	PM	drought *
	B4FA32	ZmPrx123	34.01	6.50	1-25	1	25	+	PM	etiolation, abscisic acid and pathogen *
	A0A1D6N9N5	ZmPrx124	37.42	4.84	1-23	2	25	---	PM	pH and sulfur deficiency *
	K7TMB0	ZmPrx128	35.80	8.05	1-36	3	30	---	MoM	---
	K7U151	ZmPrx129	33.75	6.80	1-26	1	20	---	PM	---
	A0A1D6JNY2	ZmPrx132	46.64	4.99	1-22	1	40	+	Vac	---
	K7VFH6	ZmPrx135	35.67	4.98	1-31	---	22	---	ER	abscisic acid and heat *
	C4IZ20	ZmPrx136	34.48	8.45	1-22	2	21	---	PM	etiolation, drought and pathogen *
	A0A1D6JF04	ZmPrx140	37.88	8.88	1-21	3	31	---	PM	pH *

^1^http://www.uniprot.org/; ^2^http://peroxibase.toulouse.inra.fr/; ^3^https://web.expasy.org/protparam/; ^4^http://www.cbs.dtu.dk/services/SignalP/; ^5^http://www.cbs.dtu.dk/services/NetNGlyc/; ^6^http://www.cbs.dtu.dk/services/NetPhos/; ^7^https://prosite.expasy.org/; ^8^http://psort1.hgc.jp/form.html.

**Table 2:** Docking results of multiple ligand molecules by Firedock (http://bioinfo3d.cs.tau.ac.il/FireDock/). Putative structures of the membrane-bound peroxidases CroPrx01 (5aog.1.A), AtPrx64 (3hdl.1.A), AtPrx47 (5twt.1.A), MtPrx02 (5twt.1.A), OsPrx95 (3hdl.1A) and ZmPrx01 (3hdl.1.A) were modeled by SWISS-MODEL (https://swissmodel.expasy.org/). Horseradish peroxidase (HRP, 1hch) was used for comparison.

Substrate	CroPrx01	AtPrx64	AtPrx47	MtPrx02	OsPrx95	ZmPrx01	HRP
ascorbic acid	−5.47	−5.39	−5.94	−5.48	−5.56	−5.29	−5.88
l-DOPA ^1^	−8.23	−8.39	0.24	12.62	7.11	12.34	−0.34
indole acetic acid	−15.41	−13.66	−12.95	−17.24	−13.05	−13.6	−11.79
NADH ^2^	−9.26	−8.6	−12.82	−12.42	−13.32	−9.89	−6.29
NADPH ^3^	−12.93	−13.69	−13.78	−11.69	−11.95	−12.49	−5.44
cinnamyl alcohol	−7.95	−7.98	−7.67	−8.74	−7.67	−6.39	−5.85
coniferyl alcohol	−4.56	−4.33	−3.77	−3.73	−4.85	−4.54	−3.81
sinalpyl alcohol	−7.31	−7.88	−5.23	−8.02	−6.51	−7.26	−3.35
ferulic acid	−6.8	−7.18	−8.14	−9.04	−6.11	−5.22	−6.86
caffeic acid	−5.35	−5.34	−5.06	−5.98	−5.91	−4.88	−2.07
*p*-coumaric acid	−4.96	−5.77	−5.32	−5.46	−6.08	−6.4	−5.54

^1^ DOPA, l-3,4-Dihydroxyphenylalanin; ^2^ NADH, Nicotinamide adenine dinucleotide; ^3^ NADPH, Nicotinamide adenine dinucleotide phosphate.

**Table 3:** Homologous and most similar sequences of membrane-bound class III peroxidases in other species. Sequence identity is given in brackets. Protein names are given according to PeroxiBase.

Protein	*Z. mays*	*O. sativa*	*M. truncatula*	*A. thaliana*	*C. roserus*
*CroPrx01*	*ZmPrx16 (55%)*	*OsPrx23 (53%)*	*MtPrx48 (54%)*	*AtPrx12 (59%)*	*CroPrx03 (84%)*
*AtPrx64*	*ZmPrx136 (65%)*	*OsPrx32 (60%)*	*MtPrx55 (72%)*	*AtPrx66(52%)*	*CroPrx40 (72%) CroPrx43 (72%)*
*AtPrx47*	*ZmPrx15 (57%)*	*OsPrx117 (60%)*	*MtPrx19 (71%)*	*AtPrx66 (47%) AtPrx64 (46%)*	*CroPrx09 (75%)*
*MtPrx02*	*ZmPrx120 (52%)*	*OsPrx40 (59%)*	*MtPrx70 (96%)*	*AtPrx52 (46%)*	*CroPrx04 (55%)*
*OsPrx95*	*ZmPrx94 (73%)*	*OsPrx97 (50%) OsPrx134 (50%)*	*MtPrx07 (43%)*	*AtPrx56 (46%)*	*CroPrx49 (48%) CroPrx59 (47%)*
*ZmPrx1*	*ZmPrx101 (68%)*	*OsPrx12 (74%)*	*MtPrx07(52%)*	*AtPrx39 (46%)*	*CroPrx14 (48%)*

## Abbreviations

AGP	Arabinogalactan protein
ARE	Antioxidant-responsive elements
AtPrx47	Arabidopsis thaliana peroxidase 47
AtPrx64	AtPer64, Arabidopsis thaliana peroxidase 64
AVLB	-3′,4′-anhydrovinblastine
BLAST	Basic local alignment search tool
BvPrx12	Beta vulgaris peroxidase 12
CASPs	Casparian strip domain proteins
CroPrx01	CrPrx1; *Catharanthus roseus* peroxidase 1
DRM	Detergent resistant membrane
ER	Endoplasmic reticulum
ESB1	Enhanced Suberin 1
Gaertn.	Gärtner, Joseph
GFP	Green fluorescence protein
hrCNE	High resolution clear native electrophoresis
Heynh.	Heynhold, Gustav,
HRP	Horseradish peroxidase
IAA	Indol-3-acetic acid
l-DOPA	l-3,4-dihydroxyphenylalanine
MEGA7	Molecular Evolutionary Genetics Analysis Version 7.0
B.May.	Bernhard. Meyer
MiM	Mitochondrial inner membrane
MoM	Mitochondrial outer membrane
MS	Mass spectrometry
MtPrx02	Medicago truncatula peroxidase 2
MW	Molecular weight
MYB36	MYB domain protein 36
NADH	Nicotinamide adenine dinucleotide
NADPH	Nicotinamide adenine dinucleotide phosphate
OsPrx95	*Oryza sativa* peroxidase 95
PCA	Pyrrolidone carboxylic acid
pI	Point isoelectric
PM	Plasma membrane
pmPOX2a	Plasma membrane peroxidase 2a
PSORT	protein subcellular localization prediction tool
PsPrx13	*Pisum sativum* peroxidase 13
PyMOL	Python-enhanced molecular graphics tool
Rboh	Respiratory burst oxidase homolouge
ROS	Reactive oxygen species
Scherb.	Scherbius, Johannes
SOD	Superoxide dismutase
TMH	Transmembrane helix
TMHMM	Trans Membrane Helix Markov Model
Vac	Vacuole
ZmPrx01	*Zea mays* peroxidase 1
